# To be wounded is to be read: disability and the structures of nonviolent conflict from Shakespeare to the Victorians

**DOI:** 10.3389/fsoc.2026.1964488

**Published:** 2026-09-23

**Authors:** Lidia Mihaela Necula

**Affiliations:** English Department, Faculty of Letters, “Dunărea de Jos” University of Galaţi, Galaţi, Romania

**Keywords:** disability studies, narrative ethics, nonviolent conflict, Shakespeare, social legibility, Victorian fiction, vulnerability

## Abstract

This article proposes *nonviolent conflict* as a conceptual framework for examining struggles organised through the social interpretation and mediation of embodied vulnerability rather than through direct physical force. Bringing disability studies into conversation with theories of vulnerability, epistemic injustice, pain, and narrative ethics, it asks how wounded or impaired bodies become sites of contested authority, credibility, care, access, and representation. Through theoretically informed comparative close readings of *Titus Andronicus, King Lear, A Christmas Carol*, and *Jane Eyre*, the article develops four interconnected modalities of nonviolent conflict: *reading and institutional legibility, care and control, access and space, and aesthetics as tactics*. The comparison does not posit a linear historical progression from early modern spectacle to Victorian regulation; instead, the four texts function as historically differentiated cases through which the conceptual model is elaborated. Lavinia foregrounds conflicts over testimony and counter-speech; Gloucester exposes the relation among impairment, mobility, dependency, and trust; Tiny Tim reveals the mediation of vulnerability through sentiment and charity; and Rochester places disability within structures of intimacy, care, space, and relational authority. Across these configurations, practices of interpretation and mediation become sites of conflict when they distribute authority, resources, participation, or belonging around embodied difference. Nonviolent conflict thus offers a transferable analytical model for examining how social legibility itself becomes contested without collapsing interpretation, care, dependency, or classification into forms of physical violence.

## 1 Introduction: disability and the narrative redistribution of conflict

Conflict in literary studies is still most frequently imagined through the language of collision: wars, rivalries, betrayals, revolutions, ideological confrontations, dramatic antagonisms. Even when criticism turns towards ethical or psychological dimensions, conflict often remains tethered to visible forms of opposition and force. Yet some of the most consequential struggles represented in literature unfold precisely where violence appears to recede. They emerge through acts of interpretation: through the allocation of credibility, the regulation of care, the organisation of access, and the struggle over who possesses the authority to define the meaning of a body. Conflict, in this sense, does not necessarily disappear when force subsides. It may instead be redistributed across interpretive, relational, and material systems.

Recent discussions in narrative ethics have emphasised the role of storytelling in organising instability into intelligible forms. Scranton (2018, p. 49) argues that narratives help individuals and societies render disorder psychologically and ethically manageable through their capacity for telling “the stories that we want to hear,” while McGlothlin (2023, p. 111) interrogates the ways narratives appear to resolve violence “in ways that fulfil the reader's expectations and produce a satisfying sense of completeness.” Meretoja (2018, pp. 14–18) similarly understands narrative as a framework through which ethical relations become imaginable and socially negotiable, while Frank's (1995) account of wounded storytelling treats bodily suffering not as mute evidence alone but as something narratively mediated through acts of self-understanding, recognition, and ethical relation.

Disability studies approaches related problems from several directions. Garland-Thomson's (2009, 2011) work on staring and misfitting locates bodily difference within relations of visibility and between bodies and environments; Mitchell and Snyder's (2000) expose disability's recurrent recruitment into narrative structure; and Davis (1995) traces the historical production of normalcy as a cultural and disciplinary category. Other strands of scholarship bring questions of pain, credibility, interpretive resources, vulnerability, and interdependence into the discussion (Scarry's, 1985; Fricker, 2007; Butler, 2020). Shakespearean and Victorian disability scholarship has likewise examined embodiment, performance, interdependence, moral recognition, narrative function, and care (Gottlieb, 2018; Williams, 2021; Holmes, 2004; Bolt et al., 2012).

Taken together, these approaches illuminate different mechanisms through which embodied difference becomes visible, narratively useful, socially accommodated, epistemically credible, or dependent upon relations of care. They do not, however, describe identical relations. Staring concerns the social organisation of looking; misfitting, the encounter between bodies and environments; narrative prosthesis, the structural use of disability within narrative; epistemic injustice, the distribution of credibility and interpretive resources; vulnerability, relations of dependency and exposure; and care, questions of assistance, autonomy, and responsibility. What remains less clearly conceptualised is how these distinct relations may operate together as a structure of conflict: how struggles over interpretation, credibility, care, access, and representation persist after direct physical force has ceased, or arise where physical force is not the principal mechanism through which authority is exercised.

This article uses *nonviolent conflict* to name this field of struggle. The term is used here in a sense distinct from its established use in civil-resistance scholarship, where nonviolent conflict commonly refers to organised collective action conducted without physical violence. The present framework does not describe a strategy of political mobilisation. Rather, nonviolent conflict designates an interpretive and relational structure in which competing claims over credibility, dependency, access, recognition, and representation are organised without requiring immediate physical coercion. This does not imply either the absence of violence or the equivalence of interpretive struggle with bodily assault. A wound may be produced by physical force, but the struggles gathering around it subsequently take other forms. Staring, testimony, assistance, charity, diagnosis, classification, accommodation, and interpretation may protect or sustain vulnerable subjects; they may also distribute authority unevenly. The question is therefore not whether these practices are secretly forms of violence, but when and how they become contested: who is believed, who is assisted, who can participate, whose interpretation becomes authoritative, and how the embodied subject can intervene in the meanings produced around them.

Disability occupies a particularly charged position within this structure because wounded or impaired bodies rarely function as passive signs of suffering alone. They become sites upon which knowledge, dependency, mobility, care, and social legitimacy are negotiated. *To be wounded in literature is also to become legible*: to enter systems of observation, interpretation, judgement, assistance, and response. Yet legibility does not necessarily produce authority. A body may be intensely visible while the person inhabiting it remains unable to determine what that visibility means. Conversely, embodied subjects may negotiate, redirect, or contest the interpretations gathered around them even when they cannot control the conditions under which they are read. The distinction between *being interpreted* and *participating in interpretation* is therefore central to the account of nonviolent conflict developed here.

The article develops this account through four interconnected modalities: *reading and institutional legibility; care and control; access and space; and aesthetics as tactics*. These are not intended as an exhaustive taxonomy of disability representation, but as recurrent sites at which embodied difference becomes socially consequential through meaning, assistance, dependency, material access, and embodied expression.

The model is tested through theoretically informed comparative close readings of four historically differentiated cases: Shakespeare's *Titus Andronicus* and *King Lear*, Dickens's *A Christmas Carol*, and Brontë's *Jane Eyre*. The works are not presented as a representative history of literary disability, nor does their juxtaposition posit a linear progression from early modern to Victorian understandings of impairment. Rather, they place different forms of embodied vulnerability within distinct narrative, spatial, and relational environments. Lavinia foregrounds constrained testimony and struggles over epistemic authority; Gloucester brings impairment into relation with mobility, spatial knowledge, dependency, and trust; Tiny Tim makes visible the mediation of vulnerability through sentiment and charity; and Rochester places acquired impairment within structures of intimacy, assistance, and relational authority. Their *canonical persistence* is methodologically useful because these figures have remained culturally legible and critically contested, allowing the model to be tested across familiar representations whose meanings cannot be treated as critically settled. The comparison is therefore conceptual rather than periodising: Shakespearean drama makes public spectatorship, bodily signs, testimony, and interpretive instability particularly visible, while the Victorian texts bring affective response, domestic care, dependency, and narrated intimacy into sharper focus. The question is not whether the same disability paradigm operates from Shakespeare to the Victorians, but whether recurrent conflicts over embodied legibility can be identified without erasing the historical and formal differences through which they emerge.

Across these cases, disability makes visible a broader social grammar in which power operates not only through force but through the practices by which bodies are read, assisted, situated, accommodated, and made recognisable. Nonviolent conflict consequently shifts attention from the wounded body as an object carrying a predetermined meaning towards the relations through which meaning acquires authority and material consequence. It asks not only *what does the wounded body mean?*, but *who is authorised to answer that question, what follows from the answer, and how far can the wounded subject participate in producing, negotiating, or contesting it?*

## 2 Theoretical framework: disability and the structures of nonviolent conflict

The conceptual problem addressed by nonviolent conflict begins with the distinction between the event of violence and the social processes through which its meaning and consequences are organised. A wound may be produced by force, but it subsequently enters relations of testimony, spectatorship, care, mobility, classification, and recognition. These relations distribute authority: they determine who may interpret suffering, whose account becomes credible, what forms of dependency are recognised or supported, which environments permit participation, and under what conditions bodily difference becomes socially intelligible. Nonviolent conflict names the contests that emerge within this second field.

The concept therefore describes interpretive, relational, spatial, and institutional struggles in which power operates without requiring immediate physical coercion. It does not collapse bodily violence into interpretive or institutional forms of power. Rather, it identifies how struggle can be redistributed through systems of recognition, classification, care, access, and representation. The contested questions concern who possesses the authority to define the meaning of a body, whose testimony counts as knowledge, which dependencies are supported, which forms of participation become possible, and how embodied subjects negotiate the categories through which others understand them.

### 2.1 Visibility, legibility, and normativity

Legibility is central to this model because recognition is never merely descriptive. Bodies marked by injury, illness, or impairment gather interpretation around them the way scars gather memory: layer upon layer of moral explanation, suspicion, sympathy, diagnosis, aestheticisation, and judgement. To become legible is to enter relations among embodied subjects, observers, caregivers, institutions, environments, and narrative forms. Such relations may enable recognition and assistance, but they may also impose meanings, regulate dependency, or restrict participation.

Garland-Thomson's work provides an important starting point for understanding this relational field. Her account of staring identifies looking not as neutral perception but as a social encounter through which bodily difference becomes meaningful: staring asks what an unfamiliar body means (Garland-Thomson, 2009, p. 3). Her concept of misfitting subsequently relocates disability from the individual body towards the encounter between embodiment and environment (Garland-Thomson, 2011, pp. 593–95). A staircase, threshold, road, stage, or domestic space can therefore participate in disabling relations by presuming particular forms of mobility, perception, or independence. Williams's (2021) account of disability and performance further complicates the assumption that bodily capacity can be understood independently of cultural and theatrical environments.

Davis (1995, pp. 23–26) extends the problem from visibility and environment to normativity. His account of the historical production of the “normal” demonstrates how apparently descriptive categories become disciplinary frameworks through which bodies are measured and classified. Bodily variation does not, from this perspective, generate conflict by itself. Conflict emerges in the relation between embodied difference and interpretive or material systems that require bodies to become intelligible according to particular expectations.

Legibility is consequently both enabling and unstable. Recognition as injured, disabled, dependent, or in need of assistance may provide access to care or accommodation. Yet recognition also raises the question of who establishes the categories through which such needs become intelligible. The problem is not that visibility or classification is inherently oppressive, but that the capacity to make a body socially readable may carry the capacity to determine what responses that body is permitted to receive.

### 2.2 Narrative use and embodied subjectivity

The distinction between a body's social legibility and the subject's participation in producing its meaning becomes particularly important within narrative. Mitchell and Snyder's (2000, p. 49) concept of narrative prosthesis the recurrent structural use of disability within literary narrative. Such narrative usefulness creates an ethical and interpretive instability: the disabled figure may become indispensable to what the narrative means while the embodied experience of that figure remains subordinate to another character's development or to the requirements of closure.

Corpus-specific disability criticism makes this tension particularly visible. Readings of *King Lear* have challenged the reduction of Gloucester's blindness to moral or perceptual insight (Gottlieb, 2018), while disability criticism of *Jane Eyre* has questioned the treatment of Rochester's blindness and amputation merely as symbolic correction or machinery for the marriage plot (Bolt et al., 2012). In both cases, disability resists reduction either to transparent metaphor or to inert bodily fact.

Nonviolent conflict builds upon this distinction by asking what happens when the disabled figure is not only interpreted but also participates in interpretation. The characters examined here cannot control all the meanings attached to their bodies, yet gesture, speech, movement, self-description, trust, and embodied improvisation allow them to alter the interpretive field in which those meanings are produced. A subject may be constrained and still communicate; dependent, and still negotiate; culturally instrumentalised, and still exceed the function assigned to them. The relevant distinction is therefore not between passivity and autonomy, but between different conditions of participation in meaning-making.

### 2.3 Vulnerability, care, and relational authority

Vulnerability introduces a related problem because embodied dependence calls for response. Butler's (2020) account of vulnerability refuses the fantasy of the self-sufficient subject by locating exposure and dependency within the conditions of social existence rather than treating them as exceptional states of weakness. Yet recognition of vulnerability does not determine in advance what an ethical response will look like. Assistance can sustain life and expand participation while also placing decisions about needs, resources, or movement partly in the hands of another.

This tension has a specific history within disability studies. Kröger (2009) observes that disability scholarship has often regarded the language of care with suspicion because disabled people have historically been represented as dependent and non-autonomous recipients of assistance, while care research has foregrounded relationships, caring labour, and interdependence. Neither autonomy nor dependency, however, adequately describes embodied social life in isolation. Assistance may be necessary without thereby eliminating agency, while its provision may remain asymmetrical without becoming inherently coercive.

*Care and control*, as used in this article, names this unstable distribution of relational authority. It does not imply that care is disguised violence. Nor does it presume that dependency constitutes a failure of autonomy. The relevant conflict concerns the terms under which assistance is organised: who identifies need, who possesses the resources or knowledge required to respond to it, how far the recipient can negotiate those terms, and what forms of participation become possible within the caring relation. The literary cases explored below place these questions within different relations of mobility, charity, intimacy, and assistance rather than reproducing a single model of care.

### 2.4 Testimony, pain, and epistemic authority

A further dimension of nonviolent conflict concerns the relation between embodied experience and socially authorised knowledge. Scarry's (1985, p. 4) account of physical pain identifies a fundamental representational difficulty: intense pain can resist and even disrupt language. Yet precisely because embodied suffering cannot always be transparently communicated, it generates interpretive activity. Wounds, gestures, silences, diagnoses, testimony, and other signs become mediating forms through which suffering is inferred and organised.

Fricker's (2007) concept of epistemic injustice clarifies the political and ethical stakes of this mediation. Testimonial injustice occurs when prejudice diminishes the credibility granted to a speaker, while hermeneutical injustice concerns unequal access to the interpretive resources through which experience can become intelligible. The problem is therefore not simply whether suffering can be expressed, but whose expression is recognised as knowledge and according to which conventions.

For disability, this distinction is crucial. An embodied subject may possess knowledge of pain, impairment, dependence, or spatial difficulty while lacking access to the authorised forms through which that knowledge becomes socially actionable. Conversely, observers, caregivers, institutions, and narratives may possess interpretive resources unavailable to the subject while lacking access to the embodied experience they attempt to explain. Nonviolent conflict emerges in the gap between these positions: between experience and authorised interpretation, between being visible and being credible, and between having a body read by others and participating in the production of the reading. Lavinia will provide the most radical test of this distinction, since the destruction of conventional speech and writing does not entail the destruction of knowledge or the end of communicative agency.

### 2.5 From conceptual problems to modalities of nonviolent conflict

Taken together, these conversations make it possible to conceptualise nonviolent conflict through four interconnected modalities: *reading and institutional legibility; care and control; access and space; and aesthetics as tactics*. They concern, respectively, the production and authorisation of meaning; the terms of participation within relations of assistance and dependency; the material conditions of mobility and participation; and forms of embodied expression through which dominant terms of legibility may be negotiated or contested.

The modalities are neither mutually exclusive nor an exhaustive taxonomy of disability representation. A diagnosis may simultaneously constitute an act of reading, a condition of access to care, and a form of institutional classification; assistance may enable mobility while redistributing authority; gesture may communicate suffering while resisting the categories through which others attempt to interpret it. Their analytical value lies precisely in making these intersections visible.

Nonviolent conflict therefore does not designate a hidden form of violence beneath every act of interpretation, care, or accommodation. Nor does every asymmetry, dependency, or act of mediation constitute conflict. The framework becomes analytically useful where embodied vulnerability is mediated through practices that distribute authority unevenly and where that distribution carries material, epistemic, spatial, or relational consequences. Mediation, asymmetry, and consequence are thus recurrent conditions of the conflicts examined here rather than a rigid formula to which every representation of disability must conform.

Conflict, in this framework, resembles less a duel than a contested process of mediation. Bodies enter relations that seek to explain, assist, classify, accommodate, narrate, or otherwise make them socially intelligible. The question connecting the modalities is therefore not whether bodies are simply read, but who acquires authority through the mediations that make them readable, what material and social consequences follow, and how embodied subjects may participate in negotiating or redirecting those consequences.

## 3 Methodology and corpus selection

The analysis proceeds through theoretically informed comparative close reading. The four literary works are treated as purposively selected, historically differentiated cases through which the modalities of nonviolent conflict can be elaborated and tested for conceptual coherence, rather than as a representative sample from which a comprehensive history of disability might be inferred. Comparison therefore serves a conceptual rather than periodising function: it identifies recurrent relations while preserving the historical and formal differences through which those relations become visible.

Close reading attends specifically to speech, syntax, gesture, bodily movement, spatial description, narrative perspective, and scenes of assistance or interpretation. These features are examined not as interchangeable signs of disability, but as forms through which knowledge, credibility, access, dependency, and relational authority are differently distributed. The method consequently asks not only what impairment signifies within a narrative, but how textual and dramatic forms determine who can interpret embodied difference, who can respond to it, and how far the embodied subject can participate in that process.

The formal difference between drama and prose is methodologically consequential. In Shakespeare, embodied meaning is produced through staged visibility, gesture, speech, silence, movement, and relations among performers and spectators; the Victorian prose texts mediate it through narration, reported speech, affective framing, domestic relation, and retrospective interpretation. Rather than providing evidence of a linear development from early modern to Victorian disability, these differences offer controlled variation through which the same conceptual questions can be tested under different representational conditions.

The four cases therefore test rather than simply illustrate the proposed framework. Lavinia, Gloucester, Tiny Tim, and Rochester do not represent equivalent versions of disabled embodiment, nor is each confined to a single modality of nonviolent conflict. The comparison asks where the modalities intersect, where they remain uneven, and where a literary case may expose the limits of the model itself. Conceptual coherence depends not upon making the cases resemble one another, but upon determining whether recurrent structures of mediation can be identified without erasing differences in embodiment, form, historical context, and relational position.

## 4 Early modern drama: the body as emblematic conflict

In early modern drama, wounded bodies rarely remain private. Injury appears publicly, theatrically, and insistently visible, demanding interpretation before witnesses both onstage and off. As Hobgood (2021) argues, early modern spectatorship frequently positions disabled embodiment within regimes of intensified visibility and interpretive scrutiny, while Williams's (2021) work on disability and performance shows how theatrical form can produce disability through shifting relations among body, stage, and audience. Wounds speak before characters do. Blood, scars, mutilation, and blindness function not merely as consequences of violence but as signs that compel collective reading.

Yet those signs do not arrive already interpreted. Physical violence produces the wound without settling its social meaning; injury initiates further contests over testimony, recognition, dependency, and interpretive authority. Within this theatrical economy, the wounded body can appear as public text, exposed to spectatorship and moral interpretation. Craik's (2007) account of early modern sensation situates bodily signs within a culture intensely invested in reading relations between outward appearance and inward states. Shakespeare both employs and destabilises this logic: bodily signs solicit interpretation without guaranteeing certainty, making the act of reading itself a potential site of conflict. Lavinia and Gloucester expose different dimensions of this process. Lavinia possesses knowledge that others need but initially cannot access; Gloucester increasingly depends upon spatial knowledge possessed by others. The distinction prevents wounded embodiment from becoming a single model of vulnerability: the first foregrounds testimony and epistemic authority, the second mobility, dependence, trust, and relational access.

### 4.1 Lavinia in *Titus Andronicus*: legibility, testimony, and constrained counter-speech

Lavinia's mutilation produces one of the clearest instances in the present corpus of conflict shifting from bodily violence to a struggle over what bodily injury can be made to mean. The rape and mutilation are acts of extreme physical violence. What follows, however, is not simply their aftermath. Deprived of her hands and tongue, Lavinia possesses knowledge that others require while the conventional means by which that knowledge might become testimony have been deliberately destroyed. Her body is intensely legible as evidence that something has happened while remaining resistant, at first, to the more precise questions of what has happened, by whom, and with what consequences.

Marcus's discovery of Lavinia makes this distinction immediately visible:

“Who is this? My niece, that flies away so fast? Cousin, a word: where is your husband? If I do dream, would all my wealth would wake me.” (Shakespeare, 1995; *Titus Andronicus*, 2.4)

The movement from “Who is this?” to “My niece” matters. Identification precedes explanation. Lavinia is first encountered as a body that Marcus must recognise; once he recognises the person, recognition generates another demand for knowledge. Who she is can be answered. What has happened to her cannot.

Marcus attempts to bridge that gap through an intensely aestheticised language of injury. Her severed hands become branches “lopped and hewed”; blood becomes a “crimson river” rising between “rosed lips”; even her breath remains

“honey breath.” (Shakespeare, 1995, *Titus Andronicus*, 2.4)

The rhetoric does more than describe mutilation. It translates bodily catastrophe into an inherited poetic vocabulary capable of making the scene speak before Lavinia herself can do so. Her body becomes readable through metaphor at precisely the moment when her conventional means of verbal self-representation have been removed.

Anderson (2021), however, complicates any reading in which Lavinia functions only as the mute object of Marcus's aestheticising speech. Lavinia remains conscious and responsive throughout the encounter: an embodied addressee whose gestures and reactions continue to matter even while Marcus possesses almost all of the spoken language. The asymmetry is therefore epistemic as much as verbal. Lavinia knows what has happened; Marcus does not. Marcus possesses recognised means of producing interpretation; Lavinia possesses the experience that interpretation attempts to reach.

Her silence is consequently not an absence of knowledge but a crisis in the relation between embodied knowledge and the conventions through which knowledge becomes socially intelligible. Fricker's distinction between testimonial and hermeneutical injustice is illuminating here, although Lavinia's position corresponds neatly to neither. She is not simply disbelieved: initially, she cannot formulate conventional testimony that might be believed or rejected. Nor is her experience wholly without interpretive resources, since the story of Philomela will provide precisely such a resource. The difficulty lies instead in access to communicative forms through which private knowledge can become shared and actionable knowledge. Her body communicates injury, but not yet accusation.

Scarry's (1985, p. 4) argues that physical suffering “actively destroys language.” Lavinia inhabits this catastrophic threshold, but Shakespeare refuses to make linguistic loss equivalent to passivity. Gesture begins to perform the work of speech, while bodily constraint requires communication to assume unfamiliar forms.

The Ovidian parallel makes this transition particularly revealing. Lavinia can be read as another Philomela before she can use Philomela to make herself read. As long as Ovid functions only as an interpretive framework supplied by others, she remains positioned within an inherited narrative precedent. When she directs attention to the story herself, however, precedent becomes resource: an available cultural narrative recruited to communicate what her wounds alone cannot disclose.

The movement culminates in the staff-writing scene:

“Heaven guide thy pen to print thy sorrows plain, That we may know the traitors and the truth!” (Shakespeare, 1995, *Titus Andronicus*, 4.1)

Lavinia takes the staff into her mouth and guides it with her stumps, identifying both the crime and its perpetrators. The scene does not restore what has been destroyed. Speech and hands do not return, and communication remains dependent upon material assistance, literary precedent, and the willingness of others to recognise an unfamiliar bodily practice as meaningful. What emerges is not the recovery of an autonomous, unimpaired speaker but an improvised relation among body, object, text, memory, and interpretive community.

Marcus's phrase

“without the help of any hand at all” (Shakespeare, 1995, *Titus Andronicus*, 4.1)

crystallises the altered bodily terms under which writing becomes possible. Lavinia does not overcome disability by reproducing ordinary writing despite impairment; she adapts the method Marcus demonstrates, turning the staff into an enabling object through which knowledge that has been present but socially inaccessible acquires a form others can recognise as testimony.

At this point, Lavinia moves from being interpreted towards participating in interpretation. The distinction should not be overstated. She does not regain control over all the meanings attached to her body, nor does communicative success erase dependency. Marcus supplies the method; Ovid supplies a cultural script; the family provides the interpretive community within which the marks become meaningful. Her agency is therefore distributed rather than sovereign. Yet distributed agency is not the absence of agency. Lavinia uses the resources available within a radically constrained situation to redirect interpretation from speculation towards accusation.

Mitchell and Snyder's (2000, p. 49) concept of narrative prosthesis complicates rather than cancels this account: Lavinia's mutilation unquestionably drives recognition, vengeance, and narrative momentum. Yet narrative instrumentalisation does not exhaust her function. She remains dependent upon others while communicating knowledge they do not possess; an object of interpretation while becoming a participant in its production.

Within the framework of nonviolent conflict, Lavinia consequently exposes the difference between visibility and epistemic authority. Her wounds make suffering visible, but visibility alone does not identify perpetrators, establish an account, or determine what others should do. Those effects require mediation. Her improvised writing has tactical force not because disability is inherently resistant, but because an alternative material practice alters what can be known and who can contribute to knowing it.

The physical violence committed against Lavinia is therefore neither diminished nor redescribed as merely interpretive. Its aftermath generates another field of struggle over testimony, credibility, and the conditions under which embodied knowledge becomes socially legible. Once the wound is visible, the question becomes: who can make it mean? Marcus's speech offers one answer; Ovidian narrative offers another; Lavinia's staff-writing intervenes with a third. She cannot prevent her body from being read, but she can, under constrained conditions, enter the process by which that reading is made consequential.

### 4.2 Gloucester in *King Lear*: blindness, spatial knowledge, and relational authority

If Lavinia's mutilation produces a conflict over testimony, Gloucester's blindness reorganises a different relation between embodiment and authority. His injury is immediately recognisable; what becomes unstable instead is his access to space and to the knowledge required to move through it. After the violence of the blinding, conflict shifts towards orientation, assistance, trust, and the uneven distribution of spatial knowledge between Gloucester and those upon whom he becomes dependent.

Shakespeare initially complicates this altered relation to perception through Gloucester's own interpretation of blindness:

“I stumbled when I saw.” (Shakespeare, 1997; 4.1)

The line appears to invite a familiar symbolic reversal in which physical blindness produces moral or inward sight: Gloucester recognises that possessing sight did not prevent him from misreading Edgar and trusting Edmund. Yet blindness does not simply replace false vision with superior insight. The material conditions of perception have changed. Gloucester must now move through spaces he cannot visually verify, rely upon descriptions supplied by others, and negotiate environments through forms of assistance. “I stumbled when I saw” therefore marks not only an irony of sight and insight, but a transition between different arrangements of knowledge.

Disability scholarship on *King Lear* has challenged precisely the reduction of impairment to individual tragedy or moral metaphor. Drawing upon Davis's concept of dismodernism, Gottlieb (2018) reads the play as exposing the fantasy of the autonomous able body and foregrounding dependency, interdependence, accommodation, and embodied vulnerability. The present argument builds upon this account but places greater emphasis on a tension within interdependence itself. Interdependence does not by itself resolve conflict. When access to an environment depends upon another person, assistance may simultaneously enable participation and redistribute authority.

After the blinding, space itself changes meaning. Roads, pathways, and the imagined cliff at Dover become terrains whose navigation depends upon guidance and trust. What had appeared as autonomous mobility becomes visibly relational: orientation may require another person, and movement becomes an activity distributed between bodies. Access is neither wholly possessed nor wholly lost; it is negotiated through embodiment, environment, assistance, and the distribution of relevant knowledge.

This relation becomes particularly visible when Gloucester first rejects the Old Man's assistance: “Away, get thee away; good friend, be gone: / Thy comforts can do me no good at all; / Thee they may hurt” yet subsequently arranges another form of guidance for himself (Shakespeare, 1997, 4.1). His dependence does not make him merely passive. Gloucester chooses Poor Tom as the person who will lead him, asks whether he knows the way to Dover, identifies his destination, and specifies the assistance he requires:

“Bring me but to the very brim of it.” (Shakespeare, 1997, 4.1)

Edgar's response, “Give me thy arm: / Poor Tom shall lead thee,” makes the resulting interdependence physically explicit (Shakespeare, 1997, 4.1). Dependence does not eliminate agency here; it changes its form. Gloucester articulates an intention and organises the assistance necessary to pursue it, while movement becomes possible through the coordination of two bodies rather than the presumed self-sufficiency of one.

Such assistance, however, is not ethically transparent. Edgar possesses visual and topographical information that Gloucester cannot independently obtain under the particular circumstances through which they move. Gloucester can request assistance, choose a destination, and continue to articulate intentions, but he cannot verify all the information upon which his movement depends. The assisting body becomes at once a means of access and a source of knowledge.

The Dover sequence makes the resulting asymmetry especially visible. Gloucester asks to be led towards a cliff from which he intends to fall. Edgar guides him while constructing the environment verbally:

“Come on, sir; here's the place. Stand still. How fearful And dizzy'tis to cast one's eyes so low!” (Shakespeare, 1997, 4.6)

The ground beneath Gloucester has not acquired the height Edgar attributes to it; what changes is Gloucester's access to knowledge about that ground. Space is therefore not a neutral setting but an interpreted environment whose meaning is partly controlled by the person able to see it. Edgar's deception is protective: he seeks to prevent his father's suicide. Yet protection requires him to withhold accurate spatial information from the person he is protecting. Assistance expands Gloucester's capacity to move while simultaneously limiting his authority over the environment through which that movement occurs.

To describe this relation as control alone would therefore miss its ethical difficulty. Edgar does not simply dominate Gloucester, and Gloucester does not simply surrender agency. One possesses spatial information the other cannot independently verify; one must decide how far to trust the knowledge upon which his action depends. Care and deception, access and restriction, agency and dependence consequently occupy the same relational structure.

The point is not that blindness renders Gloucester incapable of knowing space. Such a conclusion would merely reproduce an ableist equation between vision and knowledge. Shakespeare offers a more complicated sensory possibility when Gloucester responds to Lear's question about whether he can “see how this world goes” with the compressed formulation,

“I see it feelingly.” (Shakespeare, 1997, 4.6)

“Seeing” is not restored here as visual perception but reconstituted through embodied sensation, experience, and affect. What Gloucester cannot independently obtain at Dover is more specific: the visual and topographical information upon which the particular action he intends depends. His vulnerability arises not from blindness in the abstract but from the relation among embodied perception, environment, intention, and distributed knowledge.

This distinction also complicates the familiar moralisation of Gloucester's blindness. His altered condition contributes to a recognition of vulnerability and material inequality, but this should not become a compensatory narrative in which disability automatically produces moral wisdom. Gloucester's suffering does not make blindness valuable because it improves him. Rather, his altered position exposes dependencies that privilege had previously allowed him to overlook. What becomes visible is not a superior disabled consciousness but the instability of the autonomous subject itself.

Gloucester consequently extends nonviolent conflict beyond interpretation in the narrow sense. Spatial access is also an epistemic relation. He remains a desiring and directing subject, but some of his intentions become executable through another body and through information another person partly controls. The resulting agency is distributed but unevenly so. His dependence does not negate agency, just as Edgar's assistance does not automatically constitute domination. Conflict emerges because access, care, knowledge, and authority become entangled within the same relation.

Gloucester thus clarifies a second configuration of nonviolent conflict. Lavinia's problem concerns the conversion of embodied knowledge into testimony; Gloucester's concerns the distribution of knowledge required for embodied action. Lavinia must find a form through which others can recognise what she knows; Gloucester must act through information and assistance that others partly control.

Blindness in *King Lear*, then, does more than reverse sight and insight. It reorganises the relations through which Gloucester inhabits space. His body becomes neither simply deficient nor newly enlightened, but differently situated within networks of assistance, knowledge, trust, and material access. The question generated by that position is not whether dependence can be eliminated, but who acquires authority when access becomes interdependent, and how the embodied subject continues to act within that distribution of authority.

### Shakespearean synthesis: from embodied knowledge to relational access

Lavinia and Gloucester do not demonstrate a single structure of wounded embodiment. The asymmetry runs in different directions. Lavinia possesses knowledge that others require but initially cannot access; Gloucester increasingly requires particular forms of knowledge that others possess. In the first case, mediation concerns the conversion of embodied knowledge into socially consequential testimony; in the second, the conversion of intention into action when spatial access becomes interdependent.

Both cases nevertheless expose a common problem: mediation enables participation while also allocating authority. Lavinia's staff-writing depends upon material and interpretive assistance; Gloucester's movement upon guidance and spatial information. Neither dependency eliminates agency, but neither produces sovereignty. Nonviolent conflict begins analytically not with dependency itself, but where the terms and consequences of mediation distribute authority unevenly: over what can count as testimony in Lavinia's case, and over what can count as actionable spatial knowledge in Gloucester's.

Theatrical form makes this distribution of authority publicly visible. Disability becomes public not simply because an impaired body appears onstage, but because theatre stages the social activity that gathers around it: staring, describing, interpreting, assisting, misreading, and responding. Lavinia is aestheticised before she is heard as a witness; Gloucester is guided through a landscape whose meaning another person partly controls. Their bodies become intensely legible, but *visibility and legibility do not guarantee epistemic or ethical authority*. The wounded body consequently functions less as a transparent emblem than as a contested public text.

The two cases also demonstrate how the modalities of nonviolent conflict intersect rather than operate in isolation. In Lavinia, reading and institutional legibility converge with aesthetics as tactics; in Gloucester, access and space intersect with care and control. What connects them is the problem of authority generated around vulnerability: who can testify, who can interpret, who can assist, and on what terms.

Shakespearean drama thus provides a particularly visible arena for examining the wounded body as a site of contested legibility. Physical violence initiates but does not settle the relations of reading, assistance, and response that follow it. The question that persists after the wound is therefore not only what has happened to the body, but who may interpret it, who may act with or on behalf of the wounded subject, and how that subject can intervene in the meanings and consequences produced around it.

## 5 Victorian fiction: affective and domestic legibility

The Victorian cases considered here bring different configurations of nonviolent conflict into view, shifting the analytic emphasis towards affective, charitable, domestic, and relational practices through which vulnerability becomes socially intelligible. Bodily difference remains visible, but visibility is increasingly entangled with questions of who provides care, how dependency is interpreted, and what forms of moral obligation vulnerability generates. As Holmes (2004) demonstrates, Victorian culture repeatedly makes physical disability a site through which sympathy, fear, dependency, and social value are negotiated. The question is therefore not simply what disability signifies, but what forms of response its representation makes imaginable.

The two cases place this problem within different economies of relation. *A Christmas Carol* makes vulnerability legible through sentiment, charity, futurity, and material dependence, while *Jane Eyre* relocates care within intimacy, economic position, assistance, and narrative authority. The distinction is not between public and private disability in any absolute sense, but between different forms through which embodied vulnerability acquires social consequence.

### 5.1 *A Christmas Carol*: sentiment, charity, and the uneasy legibility of vulnerability

Tiny Tim presents a different configuration of nonviolent conflict. Unlike Lavinia and Gloucester, he does not enter the narrative through an event of represented bodily violence. His impairment already belongs to the social and domestic world in which the reader encounters him. Conflict consequently emerges less through the aftermath of injury than through the meanings attached to visible vulnerability: pity, innocence, futurity, dependency, and the obligation to care. His body becomes legible within a sentimental economy in which vulnerability acquires moral force by eliciting feeling from others.

Dickens introduces Tim through a remarkably economical set of bodily signs: he bears a “little crutch” and has

“his limbs supported by an iron frame.” (Dickens, 2001, Stave Three)

The description offers no diagnosis and little sustained account of bodily experience. Instead, these objects make impairment immediately recognisable while leaving its medical specificity unresolved. What the narrative develops more fully is the social meaning of that visibility: Tim is carried by his father, cared for within the family, imagined as vulnerable to death, and incorporated into Scrooge's moral transformation.

Rodas's (2004) reading is useful precisely because it resists reducing Dickensian disability either to sympathetic representation or to straightforward objectification. Her account of the disabled body as a locus around which other identities and relations are organised is particularly pertinent to Tim. Mitchell and Snyder's (2000) narrative prosthesis sharpens the structural risk: disability may acquire narrative importance because it performs work for another character's development. Tim's vulnerability intensifies Scrooge's moral awakening and gives economic indifference an embodied and emotionally apprehensible form.

The possibility of Tim's death consequently exerts sentimental leverage upon Scrooge's moral imagination. Yet sentiment is not merely a substitute for material consequence. Scrooge's transformation alters the distribution of resources around the Cratchit family: money, food, security, and assistance matter, and the novella insists that changed relations of responsibility can affect bodily futures. Feeling becomes consequential when it alters material relations.

This is where *care and control* become difficult to separate. Charity is not ethically empty, nor is it merely disguised domination. Tim's access to a more secure future nevertheless depends partly upon the transformation of a wealthier man who possesses resources the Cratchits do not. Care expands material possibility while preserving an asymmetry between the person whose vulnerability generates the appeal and the person empowered to convert recognition into redistribution.

To leave Tiny Tim here, however, would reproduce the very representational structure under examination: he would remain primarily the object through which Scrooge learns to feel. His most memorable utterance permits a more cautious account of his own discursive position:

“God bless us, every one!” (Dickens, 2001, Stave Three)

The line is easily absorbed into the sentimental construction of Tim as innocent, pious, and morally exemplary. Yet its grammar matters. Tim speaks in the first-person plural. “Us” does not isolate the disabled child as the sole object of compassion, while “every one” extends the blessing beyond himself. He speaks from within the community he blesses rather than simply occupying the position of the person for whom care or blessing is requested.

This does not establish that Tim is “comfortable” with disability, nor does the novella provide sufficient access to his interior life to sustain such a claim. He is not speaking about impairment or declaring independence from care. The more limited point concerns discursive position: for a moment, he becomes the grammatical source of an inclusive ethical gesture rather than its passive recipient.

A second moment complicates his position further. Returning from church, Bob reports that Tim hoped people had noticed him “because he was a cripple,” since the sight might remind them at Christmas of the one

“who made lame beggars walk, and blind men see.” (Dickens, 2001, Stave Three)

The passage gives Tim a form of self-interpretation: he recognises that his body attracts attention and imagines that visibility as capable of producing meaning for those who see him. Yet the moment is doubly mediated. His words reach the reader through Bob, while the meaning attributed to bodily visibility remains embedded within a Christian narrative of disability, healing, and moral remembrance. Tim participates in producing his legibility without controlling the cultural terms through which that legibility becomes intelligible.

The distinction between sentimental object and speaking subject therefore cannot become absolute. Tim remains heavily mediated by narrator, family, Scrooge, the Ghosts, and conventions surrounding the vulnerable child. His voice cannot simply be extracted from those structures and redescribed as autonomous resistance. Yet neither is he exhausted by the function assigned to his body.

The closing assurance that Scrooge became “a second father” to Tiny Tim, “who did not die” (Dickens, 2001, Stave Five), crystallises this ambiguity. Tim's survival provides narrative evidence of Scrooge's transformation, and his life therefore participates in another character's moral closure. Yet survival is also a material outcome. The problem is not that care succeeds, but that the route to care depends upon an economy in which vulnerability must first become morally compelling to someone with the power to redistribute resources.

Tiny Tim consequently makes visible a form of nonviolent conflict organised through sentimental legibility. The relevant struggle concerns which forms of vulnerability become recognisable as deserving care, what kinds of feeling produce material response, and who retains authority over that response. His blessing and his reported reflection on being seen do not amount to a rejection of charity or a declaration of independence from care. They show something more limited but analytically important: a figure recruited into another character's moral transformation can still occupy a speaking and interpretive position within the economy that makes him legible.

Tiny Tim is therefore neither simply the passive object of sentimental pity nor a resistant subject standing outside that economy. He inhabits it unevenly. Nonviolent conflict lies within this unevenness: between being made to signify and participating in signification, between receiving care and entering the moral language through which care is imagined, and between the material value of assistance and the asymmetrical conditions under which assistance becomes available.

### 5.2 *Jane Eyre*: disability, care, and the redistribution of relational authority

Rochester presents a different problem from the three cases considered thus far. His acquired blindness and amputation do not primarily produce a crisis of testimony, as with Lavinia, nor a dependence upon spatial knowledge comparable to Gloucester's, nor the sentimental economy through which Tiny Tim becomes morally legible. Instead, disability enters an intimate relationship already structured by substantial inequalities of gender, wealth, age, employment, experience, and social authority. The relevant question is therefore not simply whether Rochester becomes dependent upon Jane, but what happens to relational authority when acquired disability enters a relationship whose terms were unequal before impairment.

Before the destruction of Thornfield, Rochester controls many of the conditions under which Jane encounters him. He is her employer and the owner of the space she inhabits; he possesses information about Bertha that Jane does not; and he repeatedly attempts to determine the terms upon which their relationship will proceed. Jane resists this asymmetry by asserting a moral and personal equality that the material structure of their relationship does not yet secure.

Rochester's impairment therefore enters a relationship already organised around contested authority. The fire changes that organisation dramatically, but injury neither erases the earlier inequalities nor simply reverses them. To describe Rochester's blindness and amputation as the punishment or diminution necessary to make him equal to Jane would reproduce precisely the symbolic use of impairment that disability criticism has challenged.

Disability scholarship on *Jane Eyre* has substantially complicated such readings. *The Madwoman and the Blindman*, edited by Bolt et al. (2012), demonstrates how persistently disability has been recruited into interpretations of punishment, dependence, gender, and marital equality, while Gabbard (2012) traces a movement from Rochester's custodial care of Bertha towards forms of caring labour that Jane encounters and later enacts in her relationship with Rochester. The present reading builds upon this history of care but asks a related question: how does care redistribute authority within the Jane–Rochester relationship without simply reversing its previous hierarchy? Dependence does not automatically produce equality, just as assistance does not automatically produce dominance.

Ferndean is crucial to this redistribution. Unlike Thornfield, it is secluded and removed from the social world in which Rochester previously exercised authority as employer, landowner, and prospective husband. Jane returns not as a governess economically dependent upon him but as a woman possessing an inheritance and the capacity to leave or remain on different terms. The alteration is therefore not located in Rochester's body alone: it emerges from the conjunction of bodily change, Jane's economic independence, the destruction of Thornfield, and a transformed domestic environment. Disability participates in the redistribution of authority, but it does not independently cause it.

This distinction becomes especially visible when Jane retrospectively describes their marriage:

“I was then his vision, as I am still his right hand.” (Brontë, 1994, p. 446)

The sentence initially appears to establish a straightforward substitution: Jane becomes what Rochester has lost. Yet she does not say that she restores his sight or hand. She describes herself relationally as “his vision” and “his right hand.” The surrounding passage makes that distribution concrete: Rochester sees landscape and books “through” Jane, while she reads to him, describes what she sees, conducts him where he wishes to go, and does what he wishes to be done. Perception and action are therefore distributed across the relation rather than simply returned to Rochester as individual bodily capacities.

This language should not, however, be romanticised. To become another person's “vision” is also to occupy a position of interpretive authority. Jane mediates aspects of an environment Rochester cannot visually verify and, crucially, she is the narrator through whom the reader encounters his altered body and their life together. Care and narration converge: Jane does not merely assist Rochester within the represented world; she possesses the retrospective authority to tell us what that assistance means.

This convergence also distinguishes Rochester from Gloucester. Both experience blindness and forms of dependence, but their distributions of authority differ. Edgar controls crucial spatial information at Dover while Gloucester remains unaware of the deception organising his experience. At Ferndean, Rochester also depends upon another's visual capacities, but Jane narratively frames that dependence as chosen intimacy rather than custodial control. The comparison is therefore not blindness against blindness, but two different arrangements of embodied dependence, knowledge, and relational authority.

The novel's most famous declaration brings narrative authority sharply into view:

“Reader, I married him.” (Brontë, 1994, p. 444)

Jane does not report that Rochester married her. “I” occupies the position of grammatical subject; “him” the object of the verb. Yet the sentence should not be treated as proof that power has simply changed hands. The reversal is grammatical and narrative before it is necessarily social. What matters more is the altered set of conditions under which Jane can say “I married him” at all: she returns voluntarily, possesses economic independence, and no longer enters marriage from the position of a dependent governess. Disability is one element within this redistribution, but not its sole mechanism.

Mitchell and Snyder's (2000) concept of narrative prosthesis remains relevant because Rochester's impairment unquestionably performs narrative work: the destruction of Thornfield and the alteration of his body help reorganise the conditions under which the marriage plot can close. Yet to make disability valuable only because it repairs that plot would reproduce the instrumental logic the concept exposes. The question is not whether Rochester must become disabled before he can become an acceptable husband, but how the novel imagines intimacy once bodily capacity, economic power, domestic space, and relational dependence have all been rearranged.

Rochester's own speech complicates any account in which his altered embodiment exists only through Jane's interpretation. He interprets his condition through languages of damage, dependence, repentance, and diminished capacity, while Jane does not always accept the meanings he assigns to himself. Rochester therefore possesses embodied and interpretive knowledge that Jane cannot replace, just as Jane possesses visual and narrative capacities that he does not share in the same form. Neither position provides complete interpretive sovereignty.

Jane later describes their relation in another revealing formulation:

“To be together is for us to be at once as free as in solitude, as gay as in company.” (Brontë, 1994, p. 446)

Freedom is not imagined here as the absence of dependency or the recovery of self-sufficiency; it is imagined within togetherness. Yet the reciprocity remains narratively mediated because Jane supplies its formulation. This does not make her account false. It makes narrative authority part of the ethical structure being analysed.

The first-person form consequently produces a final asymmetry: Rochester may possess a voice within the story, but Jane possesses the story. She selects the scenes through which his impairment becomes intelligible, describes the assistance she provides, and interprets the emotional quality of their marriage. Even Rochester's partial recovery of sight reaches us through her narration. That recovery further complicates any interpretation in which permanent disability exists merely to equalise the couple: after 2 years he recovers some sight in one eye and can again navigate without being led by the hand. If permanent blindness were required simply to reduce Rochester to Jane's level, its partial reversal would disturb the supposed solution. Instead, the marriage persists across changing capacities.

Rochester's case therefore makes visible a form of nonviolent conflict organised around relational and narrative authority. Care operates alongside Jane's economic independence, her freedom to leave or remain, Rochester's changed embodiment, and the altered spatial conditions of Ferndean. These factors redistribute authority without producing perfect symmetry. A disabled subject may participate in interpreting their own condition and still lack control over the narrative in which that interpretation is preserved: Rochester speaks about his altered body, but Jane ultimately authors the account through which its place within their shared history becomes coherent.

The result is neither a narrative in which disability simply produces equality nor a reversal in which Jane becomes powerful because Rochester becomes powerless. It is a more unstable redistribution of relational authority. Rochester remains a speaking and interpreting subject; Jane is economically independent, voluntarily present, materially capable of care, and narratively authoritative. Their marriage is made possible not by the disappearance of asymmetry but by its reorganisation. Nonviolent conflict at Ferndean therefore concerns not simply who cares for whom, but who determines the terms of care, who interprets what dependency means, and who possesses the authority to tell the story in which that relation becomes legible.

### Victorian synthesis: care, dependency, and the authority to define relation

Tiny Tim and Rochester shift the framework towards forms of vulnerability mediated through care, intimacy, and material resources. Their situations are markedly different: Tim's impairment is not the consequence of represented violence, while Rochester's acquired disability enters a relationship already structured by inequalities of gender, wealth, employment, and knowledge. What connects them is not a shared form of impairment but the question of what happens when capacities or resources possessed by another person become consequential to the disabled subject's future.

In *A Christmas Carol*, authority is substantially material: Scrooge possesses resources capable of altering the conditions of Tim's life. In *Jane Eyre*, authority is differently distributed: Jane can provide assistance, but she also possesses economic independence and the retrospective narrative authority through which the Ferndean relationship becomes intelligible to the reader. Economic and narrative power are not equivalent, yet both demonstrate that care operates within distributions of capacity and authority rather than outside them.

The difference between Tim and Rochester remains crucial. Tim's participation is limited and mediated: his speech and reported interpretation of being seen complicate a sentimental structure largely organised around him. Rochester possesses considerably greater discursive presence and capacity for self-interpretation, yet Jane ultimately possesses the narrative form through which his disability and their marriage are preserved. Neither case therefore offers complete control over legibility; each reveals a different condition of participation within an uneven relation.

This unevenness does not make care inherently coercive. Tim materially benefits when Scrooge's altered recognition changes the distribution of resources, just as intimacy at Ferndean is conditioned by Jane's economic independence, voluntary return, and capacity to assist Rochester. Care can be enabling without being symmetrical, and dependency can be real without exhausting agency. The ethical question is therefore not whether dependency exists, but how far the person receiving care can participate in defining the relation through which care is provided and the meanings attached to that dependency.

The Victorian cases consequently extend the framework from the recognition of vulnerability towards the authority to respond to it. Tiny Tim asks who possesses the resources to answer recognised vulnerability, and under what affective conditions those resources are mobilised; Rochester asks who possesses the authority to define a caring relation when dependency, intimacy, and narrative control become intertwined. In both cases, care becomes ethically consequential not because dependency should disappear, but because the terms through which dependency is recognised, supported, and interpreted remain open to unequal participation.

## 6 Discussion: testing the modalities of nonviolent conflict

The four cases do not demonstrate that disability produces a single structure of conflict. Their comparative value lies in showing something more limited and more transferable: embodied vulnerability repeatedly becomes consequential through mediations connecting bodies to interpretation, resources, environments, and other people. What changes from case to case is the location of the asymmetry and the form of authority produced through it.

The four modalities–*reading and institutional legibility; care and control; access and space; and aesthetics as tactics* –should therefore be understood as analytical coordinates rather than stages or categories into which individual characters can be placed exclusively. Their intersections vary across texts and contexts. Lavinia's testimony is simultaneously a problem of legibility, access to communicative resources, and embodied expression; Gloucester's mobility connects access to assistance and epistemic authority; care in the Victorian cases is inseparable from material resources, representation, and narrative position. The modalities become useful precisely where their boundaries intersect.

Across these differences, one distinction becomes especially important: legibility does not entail authority. Disabled bodies may become available to interpretation before the subjects inhabiting them can determine what that interpretation means or what consequences follow from it. Yet the comparison also prevents a simple opposition between external interpretation and an inaccessible internal truth. The four characters participate unevenly in producing meaning around their own embodiment. The relevant distinction is therefore not between subjects who are interpreted and subjects who interpret themselves, but between different conditions of participation in processes through which interpretation becomes socially consequential.

The same qualification applies to care. *Care and control* names a tension rather than an equation. Assistance may expand the recipient's capacity to communicate, move, survive, or participate while also giving another person authority over knowledge, resources, or the terms under which assistance occurs. Asymmetry alone does not make care oppressive, just as dependency does not eliminate agency. Nor should autonomy function as the unexamined measure of successful participation. None of the four cases becomes meaningful only when dependency disappears: Lavinia's communication remains mediated; Gloucester's movement becomes interdependent; Tim's future is affected by resources he does not control; Rochester's intimacy incorporates practical assistance. Agency appears instead as situated participation within relations that may enable action while remaining unequal.

Access demonstrates equally clearly why the modalities cannot be separated. It may be material, but it may also be epistemic. Participation depends not only upon whether a body can enter or move through an environment, but upon information, assistance, resources, and the social arrangements through which that environment becomes usable. Gloucester makes this relation particularly visible, but the principle is broader: access emerges through encounters among embodiment, environment, knowledge, and other people. Conflict becomes analytically visible not in assistance itself but where the terms through which access is provided cannot be equally determined, verified, or contested.

*Aesthetics as tactics* requires a stronger qualification. It is the least evenly distributed of the four modalities. Lavinia provides its clearest instance because an altered embodied practice directly changes what can become communicable and actionable; the other cases offer weaker or differently configured forms of expressive negotiation. Disability should therefore not be romanticised as inherently resistant, nor should unconventional embodiment automatically be described as tactical. The modality applies where an embodied or aesthetic practice alters the conditions under which meaning can be produced, recognised, or acted upon.

Taken together, the cases refine the conditions under which nonviolent conflict becomes analytically useful. Three recurrent elements emerge: mediation, asymmetry, and consequence. First, some mediating practice connects embodied experience or capacity to the social world: language, gesture, assistance, representation, spatial knowledge, economic resources, classification, or narrative form. Second, that mediation distributes authority unevenly: one participant may possess greater credibility, interpretive resources, knowledge, money, mobility, or narrative control. Asymmetry alone does not establish oppression or conflict. Third, that distribution must have consequences for what can be known, communicated, accessed, received, or done.

These conditions also establish the boundary of the concept. *Nonviolent conflict* is not a synonym for every instance of interpretation, dependency, asymmetry, or social relation. Not every act of looking constitutes conflict; not every act of care constitutes control; not every representation of disability functions tactically. Nor does the framework redescribe physical violence as merely interpretive. It identifies a field in which struggle persists after violence or arises where force is not the primary mechanism through which authority operates: through consequential contests over credibility, assistance, access, recognition, representation, and participation.

The comparison consequently modifies the question of disabled agency. None of the four characters possesses complete authority over the meanings attached to their bodies, yet none is adequately described as a passive surface for the interpretations of others. Participation, rather than independence, is therefore the more useful term for the framework developed here. The relevant question is not whether an embodied subject escapes mediation, but how far they can participate in the processes through which meanings, resources, spaces, and relationships are organised, and what follows when that participation is constrained.

The framework is transferable only under similarly specified conditions. Contemporary practices of diagnosis, documentation, assessment, classification, accommodation, and eligibility also mediate between embodied experience and socially actionable recognition. Such mediation may be necessary for resources or support to become available while raising questions about who authorises categories, whose accounts become credible, and whether recognised forms accommodate lived experience. The literary cases do not provide direct evidence about contemporary administrative, medical, educational, or legal systems; the transfer is conceptual rather than evidentiary. They supply questions about mediation, authority, and participation that may be brought to other contexts rather than answers that can simply be exported to them.

The model can therefore be translated into a set of analytical questions: What form of mediation makes embodied experience socially legible? Who controls or authorises that mediation? What resources, spaces, or forms of recognition depend upon it? How far can the embodied subject participate in defining the terms through which they become legible? What material or relational consequences follow when competing interpretations are unequally authorised?

To be wounded is therefore not only to become readable. Within the framework of nonviolent conflict, the more fundamental question is how legibility is produced, who acquires authority through its production, what consequences follow, and how the embodied subject can participate in or contest the terms of being read. The four cases establish no stable meaning of disability across historical periods. What persists is a recurrent problem of mediation: when embodied vulnerability becomes socially consequential, who acquires the authority to make that mediation consequential, and how far can the embodied subject participate in determining what follows?

## 7 Conclusion: legibility after violence

This article has proposed *nonviolent conflict* as a conceptual framework for examining forms of struggle organised through the social interpretation and mediation of embodied vulnerability. A wound may be inflicted through force, but its consequences continue to be negotiated through practices of reading, care, access, classification, and representation. The argument is therefore not that violence simply continues under another name, but that embodied vulnerability enters relations through which authority, resources, credibility, and participation remain open to negotiation.

Across the four cases, disabled subjects emerge as neither passive surfaces upon which meaning is written nor sovereign interpreters capable of determining the terms on which their bodies become legible. Lavinia redirects interpretation without controlling the conditions under which her testimony becomes intelligible; Gloucester continues to formulate intentions while acting through relational access; Tiny Tim speaks and interprets from within a sentimental economy he does not organise; Rochester interprets his altered embodiment within a relationship whose final narrative form remains Jane's. What connects these positions is not independence, but uneven participation in the processes through which embodied difference acquires consequence.

This distinction has implications beyond the literary cases without making them direct evidence for contemporary disability practices. Present-day relations of care, accommodation, classification, documentation, assessment, and access also depend upon mediations through which embodied experience becomes socially actionable recognition. The framework does not determine in advance whether any particular practice is enabling or restrictive. Instead, it directs attention towards the distribution of authority within such practices: whose account becomes credible, which forms of embodied experience fit recognised categories, what resources or forms of participation depend upon that recognition, and how far embodied subjects can participate in defining the terms through which their needs become legible. Legibility matters because recognition does not remain at the level of meaning: it can become a condition of access to resources, assistance, credibility, and participation. The transfer from the literary cases is therefore conceptual rather than evidentiary.

The framework remains necessarily provisional. Four canonical texts cannot establish its full historical or cultural range, and the concentration here on visible physical impairment constitutes a significant limitation. Cognitive, sensory, psychiatric, fluctuating, chronic, and non-apparent disabilities may involve substantially different relations among embodiment, visibility, credibility, access, and participation. Other periods, genres, and cultural contexts may likewise expose forms of mediation that require the present modalities to be revised or expanded. Nor should disability itself become a metaphor for conflict detached from the material and historically specific conditions of disabled embodiment. The value of the framework therefore depends not upon its universal application, but upon its capacity to remain revisable when confronted with bodies, environments, and relations that the present corpus does not contain.

What literature contributes to this inquiry is an unusually concentrated view of mediation itself. Narrative makes visible not only wounded or vulnerable bodies, but the networks of interpretation that gather around them: witnesses who read, caregivers who assist, environments that enable or restrict, social structures that classify or distribute resources, and embodied subjects who comply with, negotiate, redirect, or resist the meanings assigned to them. Literature can therefore make perceptible the interval between a body becoming visible and a reading of that body becoming consequential—an interval in which interpretation may become testimony, recognition may become assistance, dependency may reorganise authority, and embodied expression may alter the terms on which meaning is produced.

This interval is where the broader significance of nonviolent conflict lies. Bodies do not become socially consequential simply because they are visible, nor do interpretations remain immaterial simply because they are not acts of physical force. Once a reading affects who is believed, assisted, accommodated, admitted, supported, or authorised to participate, interpretation enters the organisation of material and relational life. The politics of legibility begins not with the fact that bodies are read, but with what readings are permitted to do.

To be wounded in literature, then, is not only to suffer or even simply to compel reading. It is to enter a field in which reading has consequences. The central question of nonviolent conflict is therefore not simply *what does the wounded body mean?* It is *who acquires the authority to make that reading consequential*, and *how far can the wounded subject participate in determining what follows?*
